# Risk factors for central lymph node metastasis in the cervical region in papillary thyroid carcinoma: a retrospective study

**DOI:** 10.1186/s12957-021-02247-w

**Published:** 2021-04-30

**Authors:** Xiang Li, Hanwen Zhang, Yu Zhou, Ruochuan Cheng

**Affiliations:** Department of Thyroid Surgery, The First Affiliated Hospital of Kunming Medical University, No. 295 Xichang Road, Kunming, 650032 Yunnan China

**Keywords:** Central lymph node metastasis (CLNM), papillary thyroid carcinoma (PTC), risk factor, retrospective study

## Abstract

**Background:**

To investigate the influence of different risk factors on central lymph node metastasis (CLNM) in the cervical region in patients with papillary thyroid carcinoma (PTC).

**Methods:**

This retrospective study included 2586 PTC patients. Potential risk factors were identified by univariate analysis, and the relationships between these factors and CLNM were ascertained by multivariable analysis. A scoring system was constructed, and the optimal cut-off value was determined.

**Results:**

On univariate analysis, sex, age, tumor diameter, multifocality, capsule invasion, vascular invasion, total number of lymph nodes in the central region, and serum thyroid peroxidase antibody (TPOAb) concentration were identified as potential risk factors for CLNM in the cervical region, whereas nerve invasion, thyroid-stimulating hormone concentration, and thyroglobulin antibody (TgAb) concentration were not. Multivariable analysis indicated that male sex, young age, large tumor diameter, multifocality, vascular invasion, a large number of central lymph nodes, and a low TPOAb concentration were significant risk factors. From these factors, a preoperative CLNM risk assessment scale was constructed for predicting CLNM in the cervical region for PTC patients.

**Conclusion:**

Male sex, young age, large tumor diameter, multifocality, vascular invasion, a large number of central lymph nodes, and a low TPOAb concentration were positively correlated with CLNM in the cervical region in PTC patients. The preoperative CLNM risk assessment scale based on these risk factors is expected to offer accurate preoperative assessment of central lymph node status in PTC patients.

## Background

Thyroid carcinoma (TC) is one of the most common malignancies in the endocrine system, and various factors have been found to contribute to its pathogenesis, such as radiation exposure, family heredity, iodine intake, sex hormone levels, and gene mutations [1]. In many countries, TC is reported to have the most rapidly increasing incidence [2–4]. According to the Chinese Cancer Statistics published in 2015 [5, 6], TC has become the seventh most common malignancy in China, with an incidence of 5.12%, and the fourth most common malignancy among Chinese women, with an incidence of 8.49%. The main pathological classifications for TC are thyroid papillary carcinoma (PTC), thyroid follicular carcinoma (FTC), thyroid medullary carcinoma (MTC) and thyroid undifferentiated carcinoma (ATC), among which PTC is the most common pathological type, accounting for 70%–80% of TC cases [7, 8]. Characterized by high differentiation, low malignancy, and slow growth, PTC has a favourable clinical prognosis with 5-year and 10-year survival rates of 95% and 90%, respectively [1, 9]. However, PTC often leads to early lymph node metastasis, with a metastasis rate of 20%–50% [10, 11]. Moreover, central lymph node metastasis (CLNM) in the cervical region has a significant influence on the recurrence rate of PTC [8]. Therefore, a method for accurate preoperative evaluation of central lymph node status is critical in the management of PTC patients.

Previous research has indicated that various factors, such as age, sex, tumor size, bilaterality, multifocality, extracapsular invasion, and angiolymphatic invasion, are significantly associated with CLNM, whereas a TC tumor location in the upper one-third of the thyroid and lymphocytic thyroiditis are believe to be protective factors agains CLNM [12]. Elevated serum thyroglobulin antibody (TgAb) and thyroid peroxidase antibody (TPOAb) levels are serological characteristics of Hashimoto’s thyroiditis (HT) [13, 14], and research has shown that the concentrations of thyroid autoantibodies induced in HT are positively correlated with the degree of thyroid tissue destruction, thyroid lymphocyte infiltration, and hypothyroidism [15]. A later study found only the TPOAb concentration to be positively correlated with the degree of thyroid lymphocyte infiltration and thyroid hypothyroidism [16]. Additional research has shown that the degree of lymphocyte infiltration in thyroid tissue is closely related to cancer cell metastasis and patient prognosis [17].

Based on this previous research, multiple risk factors are believed to be closely related to CLNM in the cervical region in patients with PTC. Therefore, the objectives of the present study were to investigate the influence of different risk factors on CLNM in the cervical region in PTC patients and to establish a CLNM risk scoring system for patients with PTC.

## Methods

### Patients

The records for all patients with PTC confirmed by postoperative pathology who were treated in the Department of Thyroid Surgery of the First Affiliated Hospital of Kunming Medical University between April 2012 and December 2018 were reviewed. The inclusion criteria for this study were: (a) complete preoperative thyroid immunological test + thyroid ultrasound or computerized tomography; (b) postoperative pathology confirming the diagnosis of PTC; (c) imaging examination indicating no distant metastasis of cancer cells; (d) treatment with surgical methods included lobectomy, isthmic resection, total/subtotal thyroidectomy + central lymph node dissection (CLND); and (e) presence or absence of CLNM in the cervical region confirmed by postoperative pathology. The exclusion criteria were: (a) a family history of other thyroid disease; (b) a history of other malignancies; (c) long-term treatment history for other chronic diseases; and (d) incomplete data.

### Data collection

Patient data were retrospectively extracted from their medical records, including demographic information, results of preoperative evaluations, surgical results, pathological data, postoperative complications, recurrence and survival information, and oncologic outcomes. According to the results of postoperative pathological examinations, the included patients were divided into the CLNM- group and CLNM+ group. CLNM- patients had no evidence of metastasis in the central lymph nodes on postoperative pathological examination, whereas CLNM+ patients had at least one central lymph node showing metastasis of carcinoma confirmed by postoperative pathology. Multifocal thyroid tumors were defined by the presence of two or more tumor lesions in the thyroid gland. The maximum diameter and number of tumors were evaluated based on pathological results combined with ultrasonic examination results. Capsule, nerve and blood vessel invasion were evaluated by pathological examination combined with intraoperative records. The protocol for this study conformed to the principles of the Declaration of Helsinki and was approved by the Ethics Committee of the First Affiliated Hospital of Kunming Medical University. All participants provided informed consent.

### Statistical analyses

SPSS version 23.0 (SPSS, Chicago, IL, USA) was used for the statistical analyses. Measurement data are expressed as mean ± standard deviation. In order to detect significant variables, a predefined P-value <0.1 was used to identify potential variables affecting CLNM in univariate analysis, and these potential variables were then included in the multivariable (logistic regression) analysis. The results of the logistic regression analysis are reported as 95% confidence intervals (CIs) and odds ratios (ORs). Standardized regression coefficients were used to quantify the discriminating power of each variable (risk factor) and to construct a scoring system to predict CLNM in the cervical region for patients with PTC. Then the receiver operating characteristic (ROC) curve was drawn, the Youden index was calculated, and the critical value corresponding to the maximum Youden index was used as the optimal cut-off value for the scoring system. Moreover, we selected 100 PTC patients who underwent thyroid surgery in the Department of Thyroid Surgery of First Affiliated Hospital of Kunming Medical University from July 2019 to December 2019 for use as a verification group in external verification for the scoring system. P<0.05 was considered to be statistically significant.

## Results

### Patient characteristics

A total of 2586 patients, including 565 males (21.8%) and 2021 females (78.2%), with PTC confirmed by postoperative pathology were retrospectively enrolled. The characteristics of these patients are presented in Table 1. The mean age of the patients was 43.01±10.82 years. Among the total study population, 74.3% of cases (n=1922) had a tumor with a maximum diameter ≤1 cm, and 60.9% of cases were single-focal carcinoma. Tumor invasiveness was relatively rare, with only 287 patients (11.1%) having capsule invasion, 69 patients (2.7%) having nerve invasion, and 110 patients (4.3%) having vascular invasion. The average serum concentrations of TSH, TPOAb, and TgAb were 2.40±1.02 uIU/mL, 201.14±398.74 uIU/mL, and 70.04±114.32 uIU/mL, respectively. A total of 982 cases had CLNM, and the other 1604 cases had no CLNM, with a cancer metastasis rate of 38.0% confirmed by postoperative pathology. The characteristics of patients in the CLNM+ and CLNM– groups are also compared in Table 1.

**Table 1:** Clinical and pathological characteristics of PTC patients

Factors	CLNM-group (*n*=1604)	CLNM+group (*n*=982)	Totalpatients/average value	%
**Gender**				
male	284 (17.7%)	281 (28.6%)	565	21.8%
female	1320 (82.3%)	701 (71.4%)	2021	78.2%
**Age (years)**	44.89±10.60	39.94±10.45	43.01±10.82	
**Tumor diameter (TD)**				
TD≤1cm	1308 (81.5%)	614 (62.5%)	1922	74.3%
1cm<TD≤2cm	245 (15.3%)	289 (29.4%)	534	20.6%
2cm<TD≤4cm	49 (3.1%)	73 (7.4%)	122	4.7%
TD>4cm	2 (0.1%)	6 (0.6%)	8	0.3%
**Tumor mulifocality**				
single-focal	1025 (63.9%)	551 (56.1%)	1576	60.9%
multifocal	579 (36.1%)	431 (43.9%)	1010	39.1%
**Capsular invasion**				
yes	158 (9.9%)	129 (13.1%)	287	11.1%
no	1446 (90.1%)	853 (86.9%)	2299	88.9%
**Nerve invasion**				
yes	38 (2.4%)	31 (3.2%)	69	2.7%
no	1566 (97.6%)	951 (96.8%)	2517	97.3%
**Vascular invasion**				
yes	25 (1.6%)	85 (8.7%)	110	4.3%
no	1582 (98.6%)	897 (91.3%)	2476	95.7%
**Number of lymph nodes in central region**	8.37±5.52	10.65±6.12	9.24±5.86	
**Thyroid function (uIU/ML)**				
TSH	2.40±1.04	2.40±1.00	2.40±1.02	
TgAb	69.21±112.28	69.92±115.80	70.04±114.32	
TPOAb	204.36±401.47	189.89±387.40	201.14±398.74	

*Abbreviations*: *CLNM* central lymph node metastasis, *TD* tumor diameter, *TSH* thyroid-stimulating hormone, *TgAb* thyroglobulin antibody, *TPOAb* thyroid peroxidase antibody

### Factors potentially associated with CLNM in PTC patients from univariate analysis

Univariate analysis revealed significant differences in sex (P<0.001), age (P<0.001), tumor diameter (P<0.001), number of lesions (P<0.001), capsule invasion (P=0.011), vascular invasion (P<0.001), total number of lymph nodes in the central region (P<0.001), and serum TPOAb concentration (P=0.078), but not nerve invasion (P=0.228), serum TSH concentration (P=0.967), and serum TgAb concentration (P=0.768) between PTC patients and without CLNM (Table 2).

**Table 2:** Univariate analysis of risk factors for CLNM among PTC patients

Factors	CLNM– group (*n*=1604)	CLNM+ group (*n*=982)	*P* values
**Sex**			<0.001
Male	284 (17.7%)	281 (28.6%)	
Female	1320 (82.3%)	701 (71.4%)	
**Age (years)**	44.89±10.60	39.94±10.45	<0.001
**TD (cm)**			<0.001
≤1 (T1a)	1308 (81.5%)	614 (62.5%)	
>1 to ≤2 (T1b)	245 (15.3%)	289 (29.4%)	
>2 < to ≤4 (T2)	49 (3.1%)	73 (7.4%)	
>4 (T3)	2 (0.1%)	6 (0.6%)	
**Tumor mulifocality**			<0.001
Single-focal	1025 (63.9%)	551 (56.1%)	
Multifocal	579 (36.1%)	431 (43.9%)	
**Capsular invasion**			0.010
Yes	158 (9.9%)	129 (13.1%)	
No	1446 (90.1%)	853 (86.9%)	
**Nerve invasion**			0.228
Yes	38 (2.4%)	31 (3.2%)	
No	1566 (97.6%)	951 (96.8%)	
**Vascular invasion**			<0.001
Yes	25 (1.6%)	85 (8.7%)	
No	1582 (98.6%)	897 (91.3%)	
**Number of lymph nodes in central region**	8.37±5.52	10.65±6.12	<0.001
**Thyroid function (uIU/ML)**			
TSH	2.40±1.04	2.40±1.00	0.967
TgAb	69.21±112.28	69.92±115.80	0.768
TPOAb	204.36±401.47	189.89±387.40	0.084

*P* values for comparisons of CLMN– and CLMN+ groups

*Abbreviations*: *CLNM* central lymph node metastasis, *TD* tumor diameter, *TSH* thyroid-stimulating hormone, *TgAb* thyroglobulin antibody, *TPOAb* thyroid peroxidase antibody

### Factors independently associated with CLNM in PTC patients from multivariable (logistic regression) analysis

Based on the results of univariate analysis, patient sex, age, tumor diameter, multifocality, capsule invasion, vascular invasion, total number of lymph nodes in the central region, and serum TPOAb concentration were included in the multivariable analysis. The results of multivariable analysis showed that the factors of male sex, young age, large tumor diameter, multifocality, vascular invasion, large number of central lymph nodes, and low serum TPOAb concentration were positively correlated with CLNM in patients with PTC (Table 3). Capsular invasion and serum TGAb concentration were not associated with CLNM in patients with PTC.

**Table 3:** Multivariable analysis of risk factors for CLNM among PTC patients

Factors	B	OR	95% CI		P values
**Sex**					0.001
Female		1.0	(reference)		
Male	0.646	1.908	1.549	2.351	
**Age**	-0.45	0.956	0.948	0.964	0.001
**TD (cm)**					0.001
≤1		1.0	(reference)		
>1 to ≤2	0.730	2.074	1.673	2.572	0.001
>2 to ≤4	0.956	2.600	1.717	3.937	0.001
>4	1.224	3.402	0.625	18.521	0.157
**Tumor multifocality**					0.001
Single-focal		1.0	(reference)		
Multifocal	0.334	1.396	1.167	1.671	
**Capsular invasion**					0.467
No		1.0	(reference)		
Yes	0.106	1.112	0.835	1.48	
**Vascular invasion**					0.001
No		1.0	(reference)		
Yes	1.808	6.095	3.758	9.886	
**Number of lymph nodes in central region**	0.066	1.069	1.051	1.086	0.001
**Serum TgAb concentration (uIU/ML)**	-0.199	0.820	0.339	1.981	0.659
**Serum TPOAb concentration (uIU/ML)**	-0.341	0.711	0.552	0.915	0.008

*Abbreviations: CLNM* central lymph node metastasis, *TD* tumor diameter; OR, odds ratio, *CI* confidence interval, *TgAb* thyroglobulin antibody, *TPOAb* thyroid peroxidase antibody

### CLNM risk assessment scale for patients with PTC

**Fig. 1:**
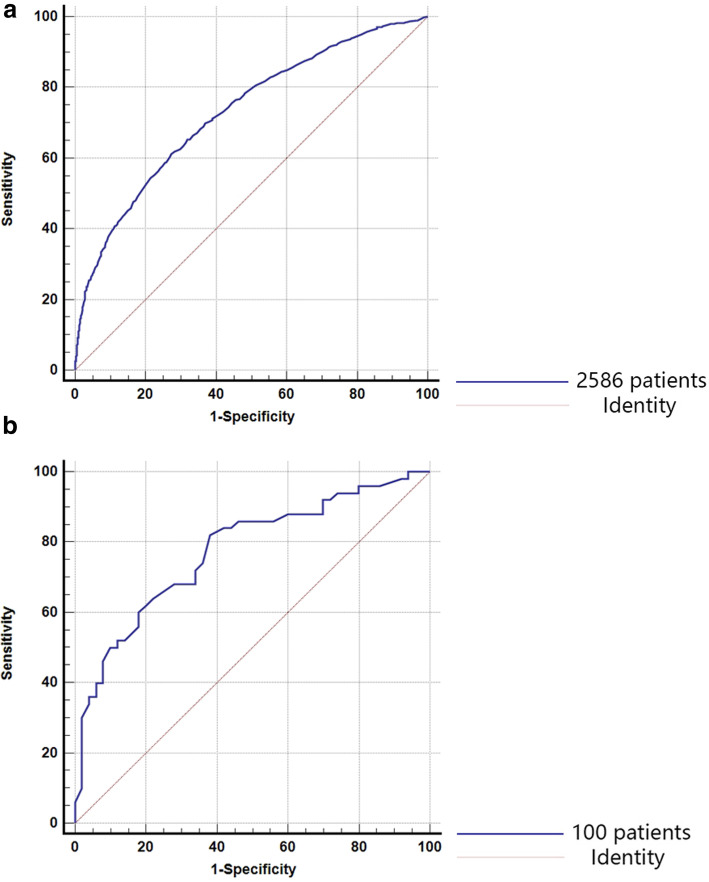
Receiver operating characteristic (ROC) curves for the ability of the CLNM risk assessment scale to predict central lymph node metastasis in the (**a**) training cohort of patients with PTC and (**b**) validation cohort of patients with PTC. The area under the curve (AUC) values for the training and validation cohorts were 0.73 and 0.775, respectively

The seven indicators identified as significantly correlated with CLNM in patients with PTC, including patient age, sex, tumor diameter, tumor multifocality, vascular invasion, total number of lymph nodes in the central region, and serum TPOAb concentration, were applied in constructing a CLNM risk assessment scale. Patient age, total number of lymph nodes in the central region, and serum TPOAb concentration were each categorized into five groups (grades) according to their quartiles. Standardized regression coefficients were used to analyze the weight of the results, and the standardized regression coefficient of 0.194 for the higher TPOAb concentration was used as the basic value for constructing the CLNM risk assessment scale. The composition of CLNM risk assessment factors for patients with PTC is shown in Table 4. The average score for the CLNM– group was 8.85±4.09, and that for the CLNM+ group was 12.85±5.06. The difference between the two groups was statistically significant (P<0.001). ROC curve analysis was conducted to evaluate the effectiveness of the CLNM risk assessment scale (Fig. 1a). The area under the ROC curve (AUC) was 0.73 (P<0.001). The most satisfactory diagnostic cut-off value for CLNM was 11.15, and the maximum Youden index was 0.338, with a sensitivity of 61.1% and a specificity of 72.7%.

**Table 4:** Composition of CLNM assessment factors for PTC patients

Factors	*P* values	OR	95% CI	B	Risk score
**Sex**	<0.001				
Male		1.87	1.516-2.307	0.626	3.2
Female		1.0	(reference)		0
**Age (years)**	<0.001				
≤35		3.788	2.935-4.890	1.332	6.9
36–42		2.118	1.641-2.734	0.75	3.9
43–50		1.552	1.196-2.015	0.44	2.3
>50		1.0	(reference)		0
**TD (cm)**	<0.001				
≤1		1.0	(reference)		0
>1 to ≤2		2.168	1.756-2.677	0.774	4.0
>2 to ≤4		2.531	1.686-3.798	0.929	4.8
>4		3.601	0.654-19.827	1.281	6.6
**Tumor mulifocality**	<0.001				
Single-focal		1.0	(reference)		0
Multifocal		1.395	1.163-1.672	0.27	1.7
**Vascular invasion**	<0.001				
Yes		6.221	3.835-10.091	1.828	9.4
No		1.0	(reference)		0
**Number of lymph nodes in central region**	<0.001				
0-5		1.0	(reference)		0
6-8		1.93	1.503-2.477	0.657	3.4
9-12		2.369	1.844-3.045	0.863	4.4
>12		2.869	2.213-3.721	1.054	5.4
**TPOAb concentration (uIU/ML)**	0.033				
Lower (≤28)		1.424	1.119-1.813	0.354	1.8
Low (>28 and ≤37)		1.326	1.007-1.747	0.282	1.5
High (>37 and ≤59)		1.214	0.938-1.572	0.194	1.0
Higher (>59)		1.0	(reference)		0

*Abbreviations*: *CLNM* central lymph node metastasis, *TD* tumor diameter, *TPOAb* thyroid peroxidase antibody

### Risk stratification based on the CLNM risk assessment scale

The most satisfactory diagnostic value of 11.15 was used as the cut-off point to divide the patients into low-risk and high-risk groups according to risk assessment scores, and the low-risk and high-risk cases in the CLNM– and CLNM+ groups were counted. The incidence of metastasis was 24.7% in the low risk group and 57.8% in the high risk group, with a significant difference between the two groups (P<0.001). The results for stratification according to the CLNM risk assessment scale are shown in Table 5.

**Table 5:** Risk stratification according to the CLNM risk assessment scale

Risk score	CLNM– group (n)	CLNM+ group (n)	Total (n)	CLNM rate
Low-risk group	1166	382	1548	24.7%
High-risk group	438	600	1038	57.8%

*Abbreviations*: *CLNM* central lymph node metastasis

### External validation of CLNM risk assessment scale

The risk assessment scale was verified through the external verification group (Table 6, Fig. 1b). The AUC for the risk assessment scale among this group was 0.775 (P<0.001), with a sensitivity of 68.0% and specificity of 72.0%. The CLNM prediction rate was 30.8% in the low-risk group and 70.8% in the high-risk group, and the difference between the groups was significant (P<0.001), providing external validation of the CLNM risk assessment scale and risk stratification.

**Table 6:** External validation of CLNM risk assessment scale

Risk score	CLNM– group (n)	CLNM+ group (n)	Total (n)	CLNM rate
Low-risk group	36	16	52	30.8%
High-risk group	14	34	48	70.8%

*Abbreviations*: *CLNM* central lymph node metastasis

## Discussion

The incidence of TC has been increasing in recent years. The main pathological type of TC, PTC, is prone to early lymph node metastasis in the central cervical region. While the mortality and recurrence rates are worrisome, surgical treatment, postoperative I^131^ treatment, and TSH inhibitory treatment can offer satisfactory efficacy [18, 19]. Keh-Chuan et al. [20] reported that the postoperative recurrence rate of PTC with CLNM in the central cervical region was 4 times than of PTC without lymph node metastasis, and the mortality rate was increased by 2.5 times. Reoperation for recurrence is associated with an increased economic burden and reduced survival rate. Therefore, a tool for accurate preoperative evaluation of lymph node status is of great significance for patients with PTC. Through our single-center study of a large cohort of PTC patients, we found that sex, age, tumor diameter, multifocality, vascular invasion, number of central lymph nodes, and serum TPOAb concentration were risk factors correlated with CLNM. Notably, capsular invasion and serum TGAb concentration were not verified to be risk factors for CLNM. From these findings, we constructed a CLNM risk assessment scale, and our validation analyses indicate that this scale can offer accurate preoperative assessment of central lymph node status in the cervical region for patients with PTC.

Many studies have shown that sex and age are independent risk factors for CLNM in patients with PTC, with male sex and younger age (<45 years) conferring a greater risk of CLNM [21–23], consistent with the results of the present study. Many thyroid tumors are found to be multifocal tumors, and the reported incidence ranges from 10%–87% [24]. Soylu et al. [25] demonstrated that the rate of CLNM positivity with multifocal PTC is statistically higher than that of those with unifocal tumors, and CLNM of multifocal PTC is significantly associated with male sex and lymphovascular invasion. The study by Ma et al. [12] verified the higher risk for CLNM with multifocal tumors. Similarly, multifocal tumor invasion was found to be a high-risk factor associated with CLNM on both univariate and multivariable analyses in our study. Together, these studies have to a considerable extent confirmed the close association between sex, age, multifocal tumor PTC, and the occurrence of CLNM, and laid a foundation for future research in managing PTC.

Previous research has shown that a large tumor size (diameter >2 cm) is significantly associated with the CLNM rate compared with a small tumor size for cN0 PTC [26, 27]. In our research, tumor size was categorized into four grades (≤1 cm, >1 to ≤2 cm, <2 to ≤4 cm, and >4 cm), according to the classification criteria for tumor size in the 8th edition of the American Joint Committee on Cancer guidelines [28]. We found that the incidence of CLNM increased with increasing tumor size, which also verifies the close correlation between tumor diameter and CLNM. The retrospective study by Falvo et al. [29] showed that histological vascular invasion can be considered a sign of increased propensity of hematogenic invasion and a corresponding increase in the relative percentage of lymphatic metastases, suggesting tumor recurrence and poor prognosis for patients with PTC. Accordingly, we agree with previous researchers that adequate preoperative evaluation, postoperative treatment, and close follow-up are necessary for these patients. Recent studies have verified that the number of central lymph nodes may be associated with CLNM, and the incidence rate of CLNM will increase with an increasing number of central lymph nodes [30]. To some extent, this also indicates that the number of central lymph nodes and some specific lymph nodes can to be used as auxiliary preoperative markers to predict CLNM. Additionally, some scholars have found that capsule invasion, nerve invasion, and serum TSH concentration are closely related to CLNM [31–34]. However, these potential associations were not observed in our current study. In order to better understand these risk factors and their significance in CLNM prevention, multicenter studies with long-term follow-up are needed.

With the increasing incidence of PTC with HT, various studies have searched for unique pathological characteristics among these patients. Some research has shown that increased levels of autoantibodies TPOAb and TgAb caused by HT may be inhibiting factors for the progression of PTC and especially favorable factors for inhibiting lymph node metastasis in the central cervical region [35, 36]. However, few studies have examined the effects of TgAb and TPOAb concentrations on CLNM in PTC patients. Therefore, we not only verified the associations of patient sex, age, tumor diameter, tumor multifocality, capsule invasion, nerve invasion, vascular invasion, number of central lymph nodes, and serum TSH concentration with PTC, but we also attempted to explore the associations between TgAb and TpoAb concentration and PTC in the present study. Our analysis identified male sex, young age, large tumor diameter, multifocality, vascular invasion, a large number of central lymph nodes, and a low serum TPOAb concentration as independent risk factors for CLNM. However, capsular invasion, nerve invasion, TSH concentration and serum TgAb concentration were not found to be significantly associated with CLNM in PTC patients. Based on the results of our study and considering the current evidence in the literature, we constructed a preoperative CLNM risk assessment scale to predict the status of the central lymph nodes for patients with PTC. We expect that our research findings and the developed risk assessment scale can provide valuable guidance and also serve as a treatment reference for PTC on the basis of current and previous studies.

Our study has some limitations: a) from this single-center cross-sectional study, the dynamic relationships between various risk factors and CLNM cannot be elucidated for patients with PTC; b) some clinicopathological features such as patient genetic mutations and PTC variants were not investigated in this retrospective analysis; and c) this study included only patients with PTC, the most common pathological type of TC, and thus, the conclusions are not applicable to FTC, MTC, or ATC. Establishment of a truly comprehensive preoperative CLNM risk assessment scale will require more data for different pathological types of TC.

## Conclusion

This retrospective study identified the factors of male sex, young age, large tumor diameter, multifocality, vascular invasion, a large number of central lymph nodes, and a low serum TPOAb concentration as positively correlated with CLNM in the cervical region for patients with PTC. However, capsular invasion, nerve invasion, TSH concentration, and serum TGAb concentration were not found to be associated with CLNM in the cervical region in PTC patients. Based on the identified risk factors, a preoperative CLNM risk assessment scale has been established to predict CLNM for PTC patients. It is expected to offer accurate preoperative assessment of central lymph node status in the cervical region and serve as a reasonable treatment reference for these patients.

## Data Availability

All data generated or analysed during this study are included in this published article.

## Abbreviations

CLNM: Central lymph node metastasis
PTC: Papillary thyroid carcinoma
HT: Hashimoto’s thyroiditis
TPOAb: Thyroid peroxidase antibody
TgAb: Thyroglobulin antibody
CLND: Central lymph node dissection
TSH: Thyroid-stimulating hormone
FTC: Thyroid follicular carcinoma
MTC: Thyroid medullary carcinoma
ATC: Thyroid undifferentiated carcinoma

## References

[1] Conzo G, Docimo G, Pasquali D, Mauriello C, Gambardella C, Esposito D, Tartaglia E, Della Pietra C, Napolitano S, Rizzuto A, Santini L (2013). Predictive value of nodal metastases on local recurrence in the management of differentiated thyroid cancer. Retrospective clinical study. BMC Surg.

[2] Siegel RL, Miller KD, Jemal A (2018). Cancer statistics, 2018. CA: a cancer journal for clinicians.

[3] Park S, Oh CM, Cho H (2016). Association between screening and the thyroid cancer “epidemic” in South Korea: evidence from a nationwide study. BMJ.

[4] Dal ML, Lise M, Zambon P (2011). Incidence of thyroid cancer in Italy, 1991-2005: time trends and age-period-cohort effects. Ann Oncol.

[5] Zheng RS, Sun KX, Zhang SW, Zeng HM, Zou XN, Chen R, Gu XY, Wei WW, He J (2019). Report of cancer epidemiology in China, 2015. Zhonghua Zhong Liu Za Zhi..

[6] Chen W, Zheng R, Baade PD, Zhang S, Zeng H, Bray F, Jemal A, Yu XQ, He J (2016). Cancer statistics in China, 2015. CA: A Cancer J Clin.

[7] Vita R, Ieni A, Tuccari G, Benvenga S (2018). The increasing prevalence of chronic lymphocytic thyroiditis in papillary microcarcinoma. Rev Endocrine Metabol Disord.

[8] Babli S, Payne R, Mitmaker E (2018). Effects of Chronic Lymphocytic Thyroiditis on the Clinicopathological Features of Papillary Thyroid Cancer. Eur Thyroid J..

[9] Seo JW, Han K, Lee J, Kim EK, Moon HJ, Yoon JH, Park VY, Baek HM, Kwak JY (2018). Application of metabolomics in prediction of lymph node metastasis in papillary thyroid carcinoma. Plos One..

[10] Lee YS, Lim YS, Lee JC, Wang SG, Son SM, Kim SS, Kim IJ, Lee BJ (2014). Ultrasonographic findings relating to lymph node metastasis in single micropapillary thyroid cancer. World J Surg Oncol.

[11] Hwang HS, Orloff LA (2011). Efficacy of Preoperative Neck Ultrasound in the Detection of Cervical Lymph Node Metastasis from. Thyroid Cancer Laryngoscope.

[12] Ma B, Wang Y, Yang S, Ji Q (2016). Predictive factors for central lymph node metastasis in patients with cN0 papillary thyroid carcinoma: A systematic review and meta-analysis. Int J Surg.

[13] Bektas Uysal H, Ayhan M (2016). Autoimmunity affects health-related quality of life in patients with Hashimoto's thyroiditis. Kaohsiung J Med Sci.

[14] Chahardoli R, Saboor-Yaraghi AA, Amouzegar A, Khalili D, Vakili AZ, Azizi F (2019). Can Supplementation with Vitamin D Modify Thyroid Autoantibodies (Anti-TPO Ab, Anti-Tg Ab) and Thyroid Profile (T3, T4, TSH) in Hashimoto's Thyroiditis? A Double Blind, Randomized Clinical Trial. Hormone and Metabol Res.

[15] Bossowski A, Urban M (2001). Serum levels of cytokines in children and adolescents with Graves' disease and non-toxic nodular goiter. J Pediatr Endocrinol Metab..

[16] Schott M, Scherbaum WA (2006). Autoimmune Thyroid disease. Deutsches Arzteblatt..

[17] Kebebew E, Treseler PA, Ituarte PH (2001). Coexisting Chronic Lymphocytic Thyroiditis and Papillary Thyroid Cancer Revisited. World J Surg.

[18] Kloos RT, Mazzaferri EL (2005). A Single Recombinant Human Thyrotropin-Stimulated Serum Thyroglobulin Measurement Predicts Differentiated Thyroid Carcinoma Metastases Three to Five Years Later. J Clinl Endocrinol Metabol.

[19] Carneiro RM, Carneiro BA, Agulnik M, Kopp PA, Giles FJ (2015). Targeted therapies in advanced differentiated thyroid cancer Cancer. Treatment Rev.

[20] Loh KC, Greenspan FS, Gee L, Miller TR, Yeo PPB (1997). Pathological Tumor-Node-Metastasis (pTNM) Staging for Papillary and Follicular Thyroid Carcinomas: A Retrospective Analysis of 700 Patients. J Clin Endocrinol Metabol.

[21] Ahn BH, Kim JR, Jeong HC, Lee JS, Chang ES, Kim YH (2015). Predictive factors of central lymph node metastasis in papillary thyroid carcinoma. Ann Surg Treatment Res.

[22] Suman P, Wang CH, Abadin SS (2016). Risk factors for central lymph node metastasis in papillary thyroid carcinoma: A National Cancer Data Base (NCDB) study. Surgery..

[23] Lee KE, Chung IY, Kang E, Koo DH, Kim KH, Kim SW, Youn YK, Oh SK (2013). Ipsilateral and contralateral central lymph node metastasis in papillary thyroid cancer: patterns and predictive factors of nodal metastasis. Head Neck..

[24] Ahn HY, Chung YJ, Kim BS, Kang KH, Seok JW, Kim HS, Park SJ, Cho BY (2014). Clinical significance of the BRAF V600E mutation in multifocal papillary thyroid carcinoma in Korea. Surgery..

[25] Soylu L, Aydin OU, Ozbas S, Bilezikci B, Ilgan S, Gursoy A, Kocak S (2016). The impact of the multifocality and subtypes of papillary thyroid carcinoma on central compartment lymph node metastasis. Eur Rev Med Pharmacol Sci..

[26] Mao LN, Wang P, Li ZY (2014). Risk factor analysis for central nodal metastasis in papillary thyroid carcinoma. Oncol Lett.

[27] Liang K, He L, Dong W, Zhang H (2014). Risk factors of central lymph node metastasis in cN0 papillary thyroid carcinoma: a study of 529 patients. Med Sci Monit..

[28] Egner J R . AJCC cancer staging manual. JAMA 2010;304:1726-1727, 15, DOI: 10.1001/jama.2010.1525.

[29] Falvo L, Catania A, D'Andrea V, Marzullo A, Giustiniani MC, de Antoni E (2005). Prognostic importance of histologic vascular invasion in papillary thyroid carcinoma. Ann Surg.

[30] Gao L, Wang J, Jiang Y, et al. The Number of Central Lymph Nodes on Preoperative Ultrasound Predicts Central Neck Lymph Node Metastasis in Papillary Thyroid Carcinoma: A Prospective Cohort Study. Int J Endocrinol. 2020;2698659. 10.1155/2020/2698659. 10.1155/2020/2698659 PMC7178523 32351558

[31] Jiao WP, Zhang L (2017). Using Ultrasonography to Evaluate the Relationship between Capsular Invasion or Extracapsular Extension and Lymph Node Metastasis in Papillary Thyroid Carcinomas. Chin Med J.

[32] Chen W, Lei J, You J, Lei Y, Li Z, Gong R, Tang H, Zhu J (2017). Predictive factors and prognosis for recurrent laryngeal nerve invasion in papillary thyroid carcinoma. Oncotargets Therap..

[33] Zhang X, Zhang X, Chang Z, Wu C, Guo H (2018). Correlation analyses of thyroid-stimulating hormone and thyroid autoantibodies with differentiated thyroid cancer. J BUON..

[34] Tam AA, Ozdemir D, Aydın C (2018). Association between preoperative thyrotrophin and clinicopathological and aggressive features of papillary thyroid cancer. Endocrine..

[35] Liang J, Zeng W, Fang F, Yu T, Zhao Y, Fan X, Guo N, Gao X (2017). Clinical analysis of Hashimoto thyroiditis coexistent with papillary thyroid cancer in 1392 patients. Acta Otorhinolaryngol Ital..

[36] Wu Q, Li Y, Wang Y (2015). Sonographic features of primary tumor as independent predictive factors for lymph node metastasis in papillary thyroid carcinoma. Clin Transl Oncol..

